# The pattern of neurocritical disorders in multicenter in Khartoum State November 2020 to January 2021

**DOI:** 10.1002/brb3.2495

**Published:** 2022-02-08

**Authors:** Khalid Mohamed Ali, Mahmoud Hussien Salih, Hiba Hassan AbuGabal, Mohammed Eltahier Abdalla Omer, Fatima Elbasri Abuelgasim Mohammed Yagoub, Ammar ElTahir Ahmed

**Affiliations:** ^1^ Faculty of Medicine Gadarif University Gadarif Sudan; ^2^ Faculty of Medicine, Department of Medicine University of Gezira Wad Madani Sudan; ^3^ Department of Internal Medicine Fajr College for Science and Technology Khartoum Sudan; ^4^ Internal Medicine Department Faculty of Medicine and Health Sciences Gadarif University Gadarif Sudan; ^5^ Faculty of Medicine University of Khartoum Khartoum Sudan

**Keywords:** ICU, neurological emergencies, neurocritical care

## Abstract

**Background:**

Neurocritical care is a growing subspecialty. It concerns with the management of life‐threatening neurological disorders. There is limited information regarding epidemiological data, disease characteristics, variability of clinical care, and in‐hospital mortality of neurocritical patients worldwide.

**Objectives:**

To study the pattern of neurocritical disorders in intensive care units.

**Methodology:**

This prospective observational study was conducted on neurocritical patients who were admitted to four intensive care units of major hospitals in Khartoum state during the period from November 2020 to January 2021.

**Results:**

Seventy‐two neurocritical patients were included in this study, 40 (55.6%) were males and 32(44.4%) were females. Twenty‐three (31.9%) patients were with stroke, 12 (16.7%) with encephalitis, 9 (12.5%) with status epilepticus, 6 (8.3%) with Guillain Barre syndrome, and 4(5.6%) with Myasthenia Gravis (MG). Twenty‐three patients (39.9%) needed mechanical ventilation (MV), which was the major indication for intensive care unit admission.

**Conclusion:**

Stroke was the dominant diagnostic pattern requiring intensive care unit admission. Mechanical ventilation was the major indication for admission. Establishing specialized neurocritical intensive care units is highly recommended.

## Graphical abstract

This prospective observational study was conducted on neurocritical patients who were admitted to four intensive care units of major hospitals in Khartoum state. Seventy‐two neurocritical patients were included in this study, 40(55.6%) were males and 32 (44.4%) were females. Twenty‐three (31.9%) patients were with stroke, 12 (16.7%), with encephalitis, 9 (12.5%) with status epilepticus, 6 (8.3%) with Guillain Barre syndrome, 4 (5.6%) with Myasthenia Gravis (MG). Twenty‐three patients (39.9%) needed mechanical ventilation (MV), which was the major indication for intensive care unit admission. Stroke was the dominant diagnostic pattern requiring intensive care unit admission. Mechanical ventilation was the major indication for admission. Establishing specialized neurocritical intensive care units is highly recommended.

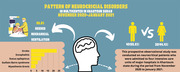

## 1 INTRODUCTION

Neurocritical care is a growing subspecialty of critical care medicine that seeks for collaboration and integration of experiences and skills between general intensivist and neurologist or neurointensivist and consistent provision of evidence‐based practice for patients with neurocritical disorders (Kramer & Guillard, 2020). A neurology ICU is an intensive care unit devoted to the care of patients with immediately life‐threatening neurological problems. Neurology ICU came into existence in response to the need for more specialized knowledge in the growing techniques to recognize and address neurological disorders. Approximately 15–20% of intensive care patients who required mechanical ventilation have an admission diagnosis involving a neurocritical care condition (Broessner et al., 2007). There is little information on the morbidity and mortality patterns of patients with neurological disorders admitted to intensive care in sub‐Saharan Africa (Adudu et al., 2007). Neurocritical care (or neurointensive care) is a medical field that concerns with the management of life‐threatening neurological disorders as well as identifying, preventing and treating secondary brain injury. Patients with neurocritical disorders that require admission to ICU constitute about 10–15% of critical care cases (Pelosi et al., 2011). In addition, many critically ill patients with sepsis or respiratory failure develop neurological complications, such as delirium, nonconvulsive status epilepticus, or neuromuscular weakness, which may in turn contribute to morbidity and an increased risk of mortality (Oddo et al., 2009). The study conducted by OP Adudu et al. from Nigeria is an interesting research due to the similarity of health facilities between Sudan and Nigeria. A total of 1124 patients were admitted to the intensive care of the hospital during the 18 years period. The duration of stay of patients in the unit ranged from 1 to 63 days. The majority of the patients (67.4%) stayed in the ICU for a week. The morbidity model showed that people with traumatic brain injuries (TBI) were in the majority (63.7%). Next come patients with severe tetanus (13.9%), hypertensive and hypoxic encephalopathy (6.4%), meningitis (4.8%), status epilepticus (3.2%), spinal cord injury (3.2%), cerebral malaria (1.3%) (Adudu et al., 2007). Kiphuth et al. (2010) conducted a retrospective study in Germany. The study investigated 796 consecutive patients who were admitted to a nonsurgical neurologic intensive care unit over a period of 2 years (2006 and 2007). They came with the following results; about 60% of all patients suffered from stroke (ischemic stroke: 31% and intracerebral hemorrhage [ICH]: 26%). Patients were diagnosed with subarachnoid hemorrhage (SAH) in 5%, epileptic seizures in 12%, meningoencephalitis in 6%, Guillain–Barré syndrome and myasthenia gravis in 3%, neurodegenerative diseases and encephalopathy in 3%, cerebral neoplasm in 3%, and intoxications in 3%. The remaining 63 patients were patients outsourced from general ICUs due to space limitations as well as patients temporarily monitored after neuroradiological procedures (Kiphuth et al., 2010). A very big amazing study named Point Prevalence In Neurocritical Care (PRINCE) was achieved in two parts sponsored by the Worldwide Organization of Neurocritical Care (Suarez et al., 2020). PRINCE was the first study to evaluate the care and patterns of neurocritical patients worldwide via performing a global survey of outcomes of neurocritical care patients. The findings concerned with the pattern of neurocritical care conditions were as follows: the most common primary reason for ICU admission was neurological monitoring (88%), and the majority of patients (42.6%) were admitted from the emergency department. The most frequent primary neurological diagnosis was SAH followed by ICH, subdural hematoma, and severe TBI (Venkatasubba Rao et al., 2020).

## 2 OBJECTIVES

### 2.1 General objective

To study the pattern of neurocritical disorders in intensive care units.

### 2.2 Specific objectives

To identify the types of neurological disorders admitted in ICUs. To highlight neurological manifestations in ICU patients who required neurological consultation.

## 3 MATERIAL AND METHODS

### 3.1 Study design

It is a prospective cross‐sectional descriptive study.

### 3.2 Study area

1. ICU in Omdurman Teaching Hospital (OTH)
2. ICU in Bashir University Hospital (BUH)
3. ICU in Ibrahim Malik Teaching Hospital (IMTH)
4. ICU in Soba University Hospital

Omdurman Hospital is one of the oldest hospitals in Sudan. It is located in Omdurman city. It is the largest hospital in the city that received patients from different states of Sudan with full‐day services. The intensive care unit of the hospital has a capacity for 10 patients.

Bashir Hospital is a full‐day university hospital. It is located in the Southern part of Khartoum city, the capital of Sudan. The intensive care unit of the hospital has a capacity of six beds.

Ibrahim Malik Hospital is located in the middle of Khartoum city. The intensive care unit of the hospital has a capacity of 6 beds.

Soba Hospital is in Khartoum city. The intensive care of the hospital has capacity of six beds.

### 3.3 Study duration

The study was conducted in the period from November 2020 to January 2021.

### 3.4 Study population

All neurocritical disorders were admitted to the ICU in addition to medical ICU patients who required neurologist consultations during their ICU stay.

### 3.5 Data collection tools and methods

The data were collected by the principal investigator. Two visits per week were conducted. A predesigned questionnaire was used to collect the data after reviewing the history, performing a clinical examination, checking records, looking for investigations and discussing with the physician in charge.

### 3.6 Plan of data analysis

Data were processed by using the computerized program Statistical Package for Social Sciences (SPSS), version 23.

### 3.7 Ethical consideration

Sudan Medical Specialization Board (SMSB) ethical committee approval was obtained.

## 4 RESULTS

Seventy‐two neurocritical patients were included in this study, 40 (55.6%) were males and 32 (44.4%) were females. Thirty‐three (45.8%) aged (18–45), 22 (30.6%) aged (46–65), and 17 (23.6%) patients were more than 65 years old. Fifty‐three (73.6%) reside in Khartoum state and 19 (24.4%) from other states. Regarding the clinical pattern of presentation: disturbed level of consciousness were found in 51 (70.8%) patients, seizures in 25 (34.7%), weakness in 41 (56%), headache in 16 (22.2%), abnormal movements in 6 (8.3%), cognitive decline in 10 (13.9%), and fever in 22 patients (30.6%). Considering clinical signs, four patients (5.6%) were deeply comatose with a Glasgow Coma Scale (GCS) of 3. Bilaterally unreactive pupils were found in 13 (18.1%), hemiparesis in 22 (30.6%) and quadriparesis in 21 (29.2%) and 5 patients (6.9%) with neck stiffness. The following investigations were frequently requested: computed tomography (CT) head for 56 (77.8%), magnetic resonance imaging (MRI) head for 29 (40.3%), cerebrospinal fluid (CSF) analysis for 18 (25%), and nerve conduction study (NCS) for 18 (25%). The pattern of diagnosis at ICU of neurocritical cases is shown in Table 1. The indications for ICU admission are shown in Table 2. Mechanical ventilation was needed for 46 (62.5%) patients. Neurological indications for MV were as follows: the failure of central regulation of respiration in 5 (6.9%) patients, inability to protect airway in 6 (8.3%), GCS less than 9 in 27 (37.5%), and impending respiratory failure in 8 (11.1%). The pattern of neurological consultations was as follows: disturbed level of consciousness (LOC) in 11 patients, seizures in 2 patients, intensive care unit acquired weakness (ICUAW) in 2 patients, and confirmation of brain death in 1 patient. Causes of disturbed LOC in consulted patients were found to be anoxic brain injury in five patients and metabolic cause in six patients. Delayed ICU admission was assumed in 13 patients (18.1%), which was considered to be due to the medical side in 6 patients and patient side in 7. The duration of ICU stay was as follows: less than 48 h for 6 (8.3%) patients, 3–6 days for 23 (31.9%), 1–8 weeks for 28 (38.9%), and 14 (19.4%) stayed more than 8 weeks. Duration on MV was as follows: less than 48 h for 7 patients, 3–6 days for 13 patients, 1–8 weeks for 23 patients, and 3 patients needed more than 8 weeks MV.

**TABLE 1:** The pattern of diagnosis at ICU of neurocritical cases

	Frequency	Percent
Stroke	23	31.9
Encephalitis	12	16.7
Status epilepticus	9	12.5
Guillain–Barré syndrome (GBS)	6	8.3
Myasthenia gravis (MG)	4	5.6
Multiple sclerosis (MS)	2	2.8
Patients needed neurological consultations	16	22.2
Total	72	100.0

**TABLE 2:** The indications for ICU admission

	Frequency	Percent
Impaired sensorium	13	18.1
Need for MV	23	39.9
Refractory status epilepticus	10	13.9
Elevated intracranial pressure	2	2.8
Medical complications	14	19.4
Monitoring	8	11.1
Treatment	2	2.8
Total	72	100.0

## 5 DISCUSSION

This study reflected the situation in Khartoum, the capital of Sudan, which has a similar practice to what is going on in most of the developing countries in which the general ICU is the main facility for neurocritical care. Starting with the distribution of the population by gender and age, this study showed no difference when compared with a large study (Venkatasubba Rao et al., 2020), which was conducted by The Worldwide Organization of Neurocritical Care (Backhaus et al., 2015). When considering the residence, it is not difficult to explain why a considerable number of neurocritical patients 19 (24.4%) came from outside Khartoum due to the fact that most of the states lack the well‐equipped ICU. Regarding the clinical pattern of presentation, there is no difference when compared to the literature review, with the predominance of clinical features like disturbed level of consciousness and seizures as well as the types’ diagnosis in which stroke was found to be the major types of condition admitted to ICU with slight variation in the studies that were conducted in the countries with established comprehensive stroke units system, the facts that were demonstrated by Howard and Kullman (2003). Encephalitis was found to have a higher burden upon ICU services when compared with the literature review (Suarez et al., 2020). This is expected in the tropics, but in 41.8% of these cases, the etiology was not confirmed which might be due to the unavailability of the facilities for full workup of viral and autoimmune encephalitis. The indications for ICU admission were compatible with international guidelines as compared to the literature review emphasized by the study of Backlachetzki et al. (2015). The study showed an obvious reduction in the number of patients admitted for monitoring or for specific therapy precautions, which I related for two reasons: first, due to very limited vacancies in general ICUs and second, because of uncommon use of such therapies particularly the unavailable one. The need for MV was the main indication for ICU admission, which was compatible with the literature review and the GCS 8 or less was the main neurological indication for mechanical ventilation as usual in similar studies (Backhaus et al., 2015). The study reflected that there was additional input from neurologists in other medical critical care patients via requested consultations, which is again going with international research. If we look at a previous study by Razvi and Bone (2003). Metabolic disorders were the main cause of disturbed LOC that necessitated neurological consultations, which was compatible with the literature review: Bleck et al. (1993) study in which metabolic encephalopathy constituted 28.6% of cases, hypoxic‐ischemic encephalopathy in 23.5% (Razvi & Bone, 2003). Delayed admission was one of the unaccepted findings particularly that which was considered to be due to the medical side, which in most cases was due to lack of vacancy or disagreement between neurologist and intensivist regarding the criteria of admission. The delayed admission from the patient side was due to two reasons either coming from outside Khartoum or due to wandering between private and governmental sectors. The duration in ICU was to some extent is similar to literature review as this is obvious when looking at what was found by Kramer and David (2013) in their study (Bleck et al., 1993). The outcome of neurocritical care management showed some differences from what was found in previous studies in the countries with a well‐established system of specialized intensive care, but some similarity with neighboring countries was noticed particularly, if we consider the mortality of each neurocritical disease.

## 6 CONCLUSION

Stroke was the dominant diagnostic pattern requiring intensive care unit admission. Neurological consultations constituted about one‐third of neurocritical conditions in the ICU. Drop‐in GCS was the main indication for admission to ICU as well as the need for mechanical ventilation. Near two‐thirds of the patients required mechanical ventilation. Delayed admission was observed due to causes distributed between the medical side and the patient side. The ICUs follow the international guidelines for admission. Establishing specialized neurocritical intensive care units with effective collaborations between neurologists, neurosurgeons, and intensivists will improve the practice in this hot area. Further studies are required in neurocritical care practice in order to highlight more areas of defects.

### PEER REVIEW

The peer review history for this article is available at https://publons.com/publon/10.1002/brb3.2495.

## References

[brb32495-bib-0001] Adudu, O. P. , Ogunrin, O. A. , & Adudu, O. G. (2007) Morbidity and mortality patterns among neurological patients in the intensive care unit of a tertiary health facility. Annals of African Medicine, 6, 174–179 10.4103/1596-3519.55701 18354942

[brb32495-bib-0002] Backhaus, R. , Aigner, F. , Schlachetzki, F. , Steffling, D. , Jakob, W. , Steinbrecher, A. , Kaiser, B. , Hau, P. , Boy, S. , Fuchs, K. , Bogdahn, U. , & Ritzka, M. , (2015) Inventory of a neurological intensive care unit: Who is treated and how long? Neurology Research International, 2015, 1. 10.1155/2015/696038 PMC4495230 26199757

[brb32495-bib-0003] Backlachetzki, S. R. , Aigner, F. , Schalachetzki, F. , Steffling, D. , Jakob, W. , Steinbrecher, A. , Kaiser, B. , Hau, P. , Boy, S. , Fuchs, K. , Bogdahn, U. , & Ritzka, M. (2015) Inventory of neurological intensive care unit: Who is treated and how long? Neurology Research International, 2015, 1. 10.1155/2015/696038 PMC4495230 26199757

[brb32495-bib-0004] Bleck, T. P. , Smith, M. C. , Pierre‐Louis, S. J. , Jares, J. J. , Murray, J. , & Hansen, C. A. (1993) Neurologic complications of critical medical illnesses. Critical Care Medicine, 21, 98–103. 10.1097/00003246-199301000-00019 8420739

[brb32495-bib-0005] Broessner, G. , Helbok, R. , Lackner, P. , Mitterberger, M. , Beer, R. , Engelhardt, K. , Brenneis, C. , Pfausler, B. , & Schmutzhard, E. (2007) Survival and long‐term functional outcome in 1155 consecutive neurocritical care patients. Critical Care Medicine, 35, 2025–2030. 10.1097/01.ccm.0000281449.07719.2b 17855816

[brb32495-bib-0006] Howard, R. , & Kullman, D. (2003) Admission to neurology admission to neurology ICU: Who, when and why. Journal of Neurology Neurosurgery and Psychiatry, 74(supp 3), iii2–9. 10.1136/jnnp.74.suppl_3.iii2 PMC1765634 12933908

[brb32495-bib-0007] Kiphuth, I. C. , Schellinger, P. D. , Köhrmann, M. , Bardutzky, J. , Lücking, H. , Kloska, S. , Schwab, S. , & Huttner, H. B. (2010) Predictors for good functional outcome after neurocritical care. Critical Care (London, England), 14(4), R136. 10.1186/cc9192 PMC2945110 20646313

[brb32495-bib-0008] Kramer, A. H. , & Guillard, P. (2020) Neurocritical care: A growing international collaborative. Neurocritical Care, 32(1), 80–83. 10.1007/s12028-019-00858-6 31571177

[brb32495-bib-0009] Kramer, A. H. , & Zygun, D. A. (2013) Declining mortality in neurocritical care patients: A cohort study in Southern Alberta over eleven years. The Canadian Journal of Anesthesia, 60, 966–975. 10.1007/s12630-013-0001-0 23877315

[brb32495-bib-0010] Oddo, M. , Carrera, E. , Claassen, J. , Mayer, S. A. , & Hirsch, L. J. (2009) Continuous electroencephalography in the medical intensive care unit. Critical Care Medicine, 37, 2051–2056. 10.1097/CCM.0b013e3181a00604 19384197

[brb32495-bib-0011] Pelosi, P. , Ferguson, N. D. , Frutos‐Vivar, F. , Anzueto, A. , Putensen, C. , Raymondos, K. , Apezteguia, C. , Desmery, P. , Hurtado, J. , Abroug, F. , Elizalde, J. , Tomicic, V. , Cakar, N. , Gonzalez, M. , Arabi, Y. , Moreno, R. , Esteban, A. , & Ventila Study Group . (2011) Management and outcome of mechanically ventilated neurologic patients. Critical Care Medicine, 39, 1482–1492. 10.1097/CCM.0b013e31821209a8 21378554

[brb32495-bib-0012] Razvi, S. S. , & Bone, I. (2003) Neurological consultations in the medical intensive care unit. Journal of Neurology, Neurosurgery, and Psychiatry, 74(Suppl 3), iii16–23. 10.1136/jnnp.74.suppl_3.iii16 PMC1765631 12933910

[brb32495-bib-0013] Suarez, J. I. , Martin, R. H. , Bauza, C. , Georgiadis, A. , Venkatasubba Rao, C. P. , Calvillo, E. , 3rd Hemphill, J. C., , Sung, G. , Oddo, M. , Taccone, F. S. , LeRoux, P. D. , & PRINCE Study Investigators (2020). Worldwide organization of neurocritical care: Results from the PRINCE study part 1. Neurocritical Care, 32(1), 172–179. 10.1007/s12028-019-00750-3 31175567 PMC7223982

[brb32495-bib-0014] Venkatasubba Rao, C. P. , Suarez, J. I. , Martin, R. H. , Bauza, C. , Georgiadis, A. , Calvillo, E. , Hemphill, J. C. , Sung, G. , Oddo, M. , Taccone, F. S. , LeRoux, P. D. , & PRINCE Study Investigators . (2020). Global survey of outcomes of neurocritical care patients: Analysis of the PRINCE study part 2. Neurocritical Care, 32, 88–103. 10.1007/s12028-019-00835-z 31486027

